# Dataset for effect of pH on caffeine and diclofenac adsorption from aqueous solution onto fique bagasse biochars

**DOI:** 10.1016/j.dib.2019.104111

**Published:** 2019-06-07

**Authors:** Yaned Milena Correa-Navarro, Liliana Giraldo, Juan Carlos Moreno-Piraján

**Affiliations:** a Grupo de investigación en Estudios Ambientales en Aguas y Suelos, Departamento de Química, Universidad de Caldas, Calle 65 No. 26-10, Manizales, Colombia; b Grupo de Calorimetría, Departamento de Química, Universidad Nacional de Colombia, Cra 30 No. 45-03, Bogotá D.C., Colombia; c Grupo de Sólidos Porosos y Calorimetría, Departamento de Química, Universidad de los Andes, Cra 1a No. 18A-10, Bogotá D.C., Colombia

**Keywords:** Remotion, Emergent contaminant, Pollutants, Agrochemical waste

## Abstract

Products of common use such as caffeine and diclofenac have been detected in surface water and groundwater, these molecules even at low concentrations have serious negative effects on animals and the environment, so they are becoming emerging contaminants. To remove pollutants from aqueous systems diverse adsorbents have been used, however materials obtained from agrochemical waste are a good alternative. This dataset present the adsorption of caffeine and diclofenac onto six fique bagasse biochars at different pH's, in addition information about textural, morphological and chemical properties of six samples of fique bagasse biochar using TGA, SEM, FTIR, PZC and Boehm's titration are provided.

**Table:** Specifications table

Subject area	*Chemistry.*
More specific subject area	*Adsorption, Surface Chemistry.*
Type of data	*Table, image, figure*
How data was acquired	*TGA–DTA (Hitachi model 7200), FTIR (Shimadzu, FR-racer-100), SEM (Tescan Lyra3),* pH meter *(Titrator SCHOT TTA20plus), spectrophotometer UV–Vis (Thermo Spectronic Genesys 5).*
Data format	*Raw and analyzed.*
Experimental factors	*Fique bagasse biochars were obtained at three different temperatures and two residence time. Moreover, data for effect of solution pH on adsorption of caffeine and diclofenac onto fique bagasse biochars are given.*
Experimental features	*Six fique bagasse biochars were evaluated for adsorption capacity of caffeine and diclofenac at different pH. These biochars were characterized by TGA, FTIR, SEM, Boehm's titration and PZC.*
Data source location	*Facultad de Ciencias, Departamento de Química, Universidad de los Andes (Bogotá, Colombia).*
Data accessibility	*Data are provided in this article.*

**Table:** 

**Value of the data** • The data of this article increase the information related to caffeine and diclofenac adsorption from aqueous solution onto biochars derived from agrochemical waste. • These data can be of great relevance for (i) other researchers working in the field of biochars and related materials worldwide and (ii) for further research in the use of waste biomass to develop biochars. • Characterization data for biochar derived from fique bagasse as a new carbonous material are given.

## 1 Data

**Fig. 1:**
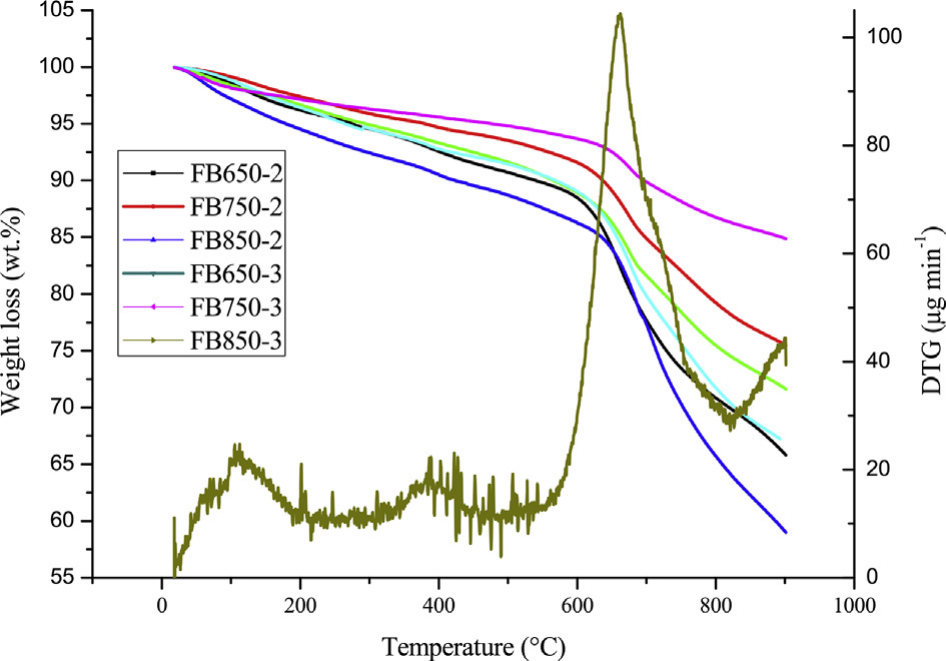
Thermograms of fique bagasse biochars.

**Fig. 2:**
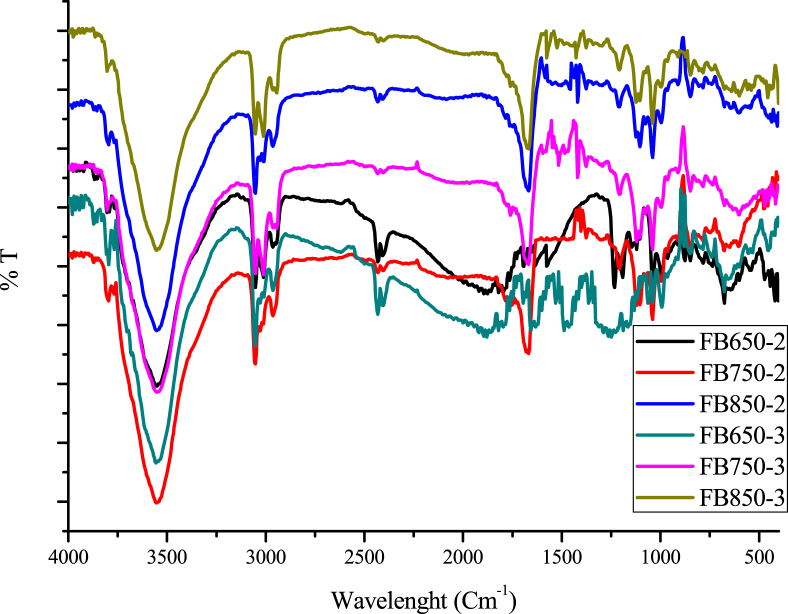
Infared spectra of fique bagasse biochars.

**Fig. 3:**
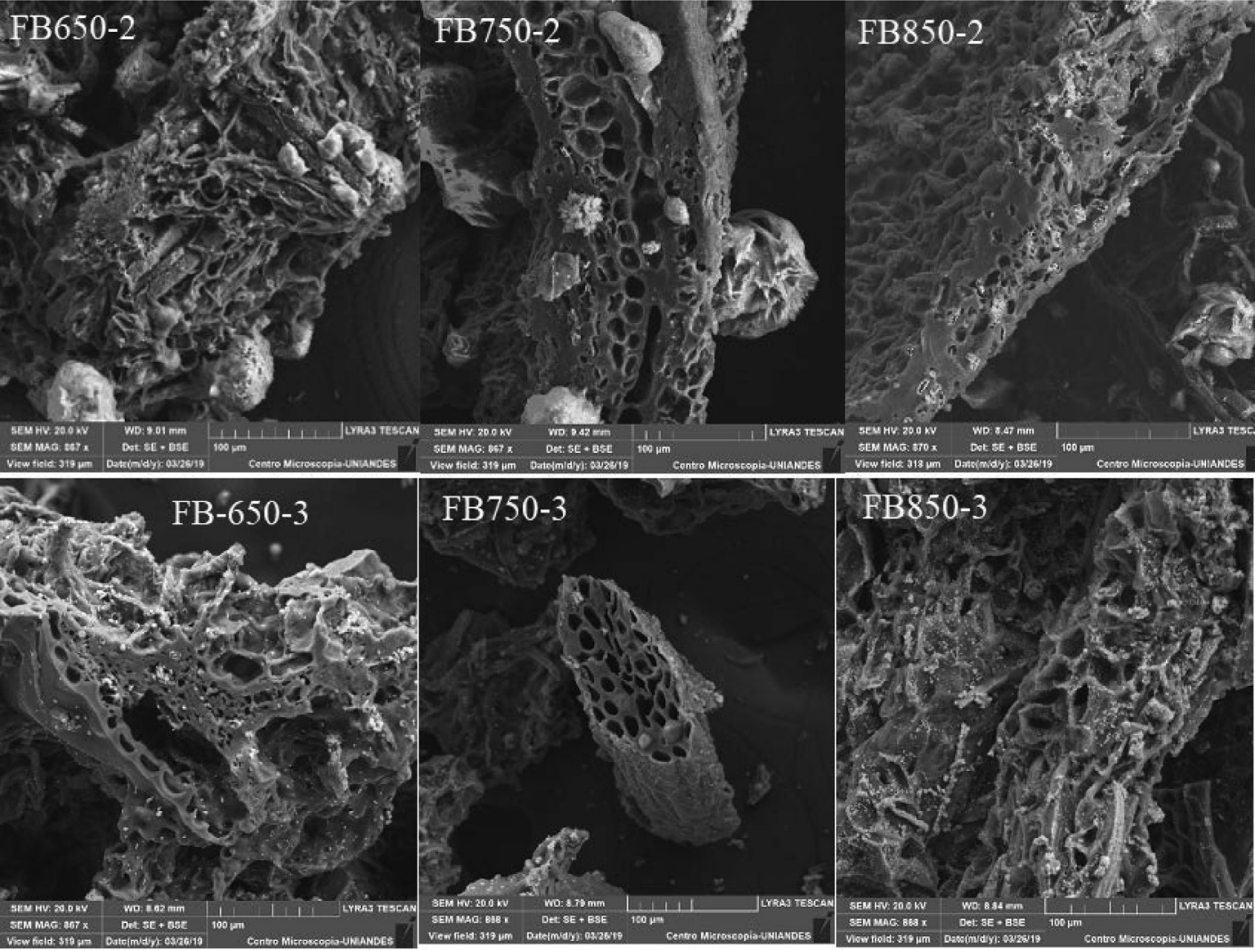
SEM images of fique bagasse biochars.

**Fig. 4:**
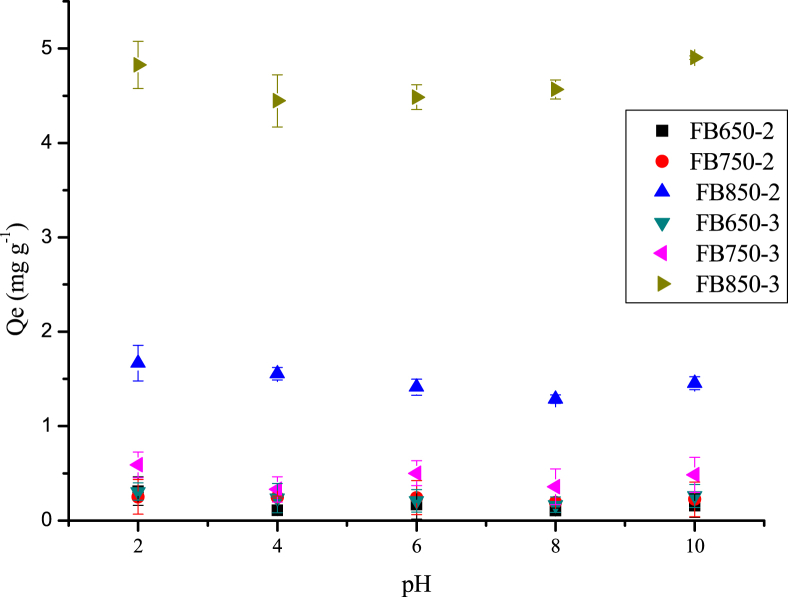
Variation of adsorption capacity of caffeine at different pH onto fique bagasse biochars (50 mg of biochars, 5 mL of 50 mg L^−1^ of CFN, 200 rpm, 20 °C, 24 h).

**Fig. 5:**
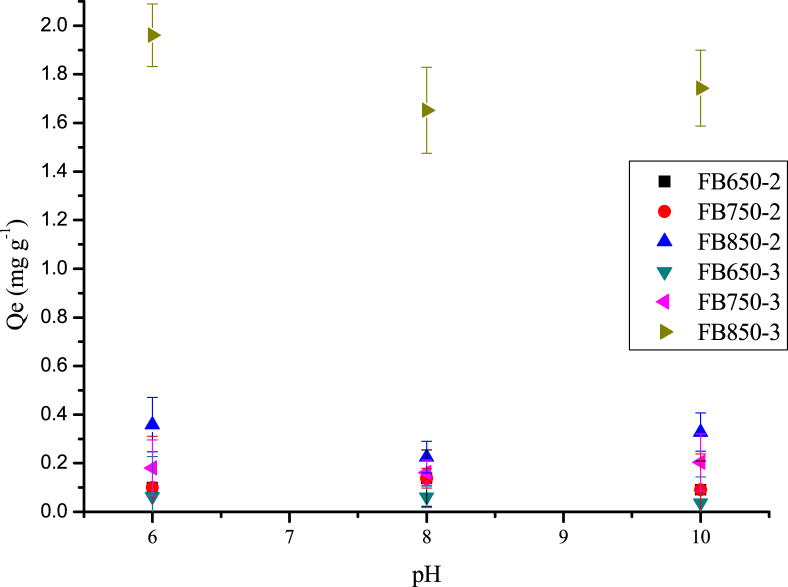
Variation of adsorption capacity of diclofenac at different pH onto fique bagasse biochars (50 mg of biochars, 5 mL of 20 mg L^−1^ of DCF, 200 rpm, 20 °C, 24 h).

Biochars were prepared from Fique bagasse (FB) collected in a farm after fiber extraction. The dataset showed characterization of biochars by thermogravimetric analysis (TGA) Fig. 1. Fourier-transform infrared spectroscopy (FTIR) Fig. 2. Scanning electron microscopy (SEM) Fig. 3. And pH effect of caffeine and diclofenac adsorption onto biochars Fig. 4 and Fig. 5 respectively. Table 1 shows the dataset of point of zero charge (PZC) and results of Boehm titrations.

**Table 1:** PZC and variation in surface acid and total basic functional groups of fique bagasse biochars.

Sample	PZC ± 0,1	Basic groups (meq g^−1^) ± SD	Carboxylic groups (meq g^−1^) ± SD	Lactonic groups (meq g^−1^) ± SD	Phenolic groups (meq g^−1^) ±SD
FB650-2	11.246	5,98 ± 0.10	0.281 ± 0.02	0.438 ± 0.01	4.67 ± 0.12
FB750-2	12.270	6.17 ± 0.08	0.228 ± 0.02	0.949 ± 0.04	3.34 ± 0.06
FB850-2	11.850	6.68 ± 0.10	0.200 ± 0.03	0.115 ± 0.04	3.34 ± 0.04
FB650-3	12.044	6.06 ± 0.06	0.309 ± 0.04	0.025 ± 0.01	1,66 ± 0.04
FB750-3	12.430	6,42 ± 0.05	0.237 ± 0.03	0.161 ± 0.06	2.53 ± 0.08
FB850-3	11.451	7.94 ± 0.06	0.142 ± 0.04	0.329 ± 0.02	1.64 ± 0.06

## 2 Experimental design, materials, and methods

### 2.1 Materials

Caffeine (CFN) and diclofenac sodium (DCF) were purchased from Merck with purity (>99%). Stock solution (1000 mg L^−1^) of CFN and DCF were prepared and then solutions of lower concentrations were obtained by dilution with distilled water and kept in darkness before the experiments were run. KBr (grade FTIR) was purchased from Panreac and the other chemical reagents used were purchased from Fisher Scientific (analytical grade).

### 2.2 Biochar preparation

The FB was dried at 100 °C for 48 h in a furnace oven. After that six types of biochars were produced by combining three temperature: 650, 750, 850 °C, and two residence time: 120 and 180 min; in the presence of nitrogen (to generate an oxygen free atmosphere) [1]. For each run heating rate was fixed at 1 °C min^−1^. These samples were coded as FB650-2, FB750-2, FB850-2, FB650-3, FB750-3 and FB850-3.

### 2.3 Biochar characterization

Thermogravimetric analyses (TGA) were conducted between 25 and 900 °C at a heating rate of 5 °C min^−1^ with nitrogen as inert purge gas at flow rate of around 100 mL min^−1^ [2]. The surface functional groups on the biochar were determined by Fourier transform infrared spectroscopy, the infrared spectra were obtained in the 400–4000 cm^−1^ wavenumber range. The samples were prepared by mixing a 0.1 mg of each biochar with 100 mg of KBr in a mortar, finally the samples were kept in an oven at 105 °C for 24 h [3]. To observe surface morphology biochar by SEM, samples were gold coated [4].

The point of zero charge (PZC) of biochars were determined by reverse mass titration. Slurries of biochar and NaCl (0.1 M) at different mass percentage were prepared. The pH of the slurries were measured after shaking at least 48 h. The PZC was determined by plotting the equilibrium pH as a function of solid weight [5]. Surface acidity and basicity of biochars were determined by Boehm's titration method. 50 mg of biochar were placed in a 50 mL vial with 0.05 N solutions of hydrochloric acid (HCl), sodium hydroxide (NaOH), sodium bicarbonate (NaHCO_3_) or sodium carbonate (Na_2_CO_3_). Each vial was closed and keep in an orbital shaker for 48 h, then 5 mL of each filtrate was titrated with NaOH and HCl, respectively [6].

### 2.4 Effect of pH on biochar adsorption capacity

Effect of pH on biochars adsorption were carried out at five different pH: 2.0, 4.0, 6.0, 8.0 and 10.0 for caffeine (CFN), and three different pH: 6.0, 8.0 and 10.0 for diclofenac (DCF); by using 50 mg of biochars with 5 mL of 50 mg L^−1^ of CFN and 20 mg L^−1^ of DCF. The samples were kept in a shaker at 200 rpm at room temperature for 24 h. After that, concentration of CFN or DCF were obtained by a calibration curve, using a Thermo Spectronic Genesys 5 spectrophotometer. All studies were carried out in triplicate. The CFN and DCF quantity adsorbed, Qe (mg g^−1^) were determined from the Eq. (1).

(1) $$Q_{e}=\frac{V\left(Co-Ce\right)\;\;}{W}$$

Where Co is the initial concentration of CFN or DCF (mg L^−1^), Ce is the concentration of CFN or DCF at equilibrium, V (L) is the volume of CFN or DCF solution and W (g) is the dry mass of Bchs tested [7].

Fique bagasse biochar thermograms show mass change between the range of 100–600 °C and graphite formation above this temperature (Fig. 1). On the other hand, FT-IR spectra of biochars evaluated have the typical bands at 3600, 2900 and 1600 cm-1, characteristic of the O–H, C–H and C=O groups (Fig. 2). Besides, the SEM images show the heterogeneous surface of biochars analyzed and the difference of developed pores (Fig. 3). In addition, the increase in temperature and residence time influence the characteristic of biochar obtained, such as the number of basic groups increased, while the concentration of acid groups decreased with the increment of pyrolysis temperature (Table 1). Finally, it was observed that FB850-3 was the one with the highest adsorption capacity of both pollutants: caffeine and diclofenac, and it was determined that the pH did not affect significantly the adsorption capacity of the evaluated biochars (Fig. 4, Fig. 5).
